# ANCA-Associated Systemic Vasculitis Presenting With Hypertrophic Spinal Pachymeningitis: A Report of 2 Cases and Review of Literature

**DOI:** 10.1097/MD.0000000000002053

**Published:** 2015-11-20

**Authors:** Xia Li, Jiuliang Zhao, Qian Wang, Yunyun Fei, Yan Zhao

**Affiliations:** From the Department of Rheumatology and Clinical Immunology, Key Laboratory of Rheumatology & Clinical Immunology, Ministry of Education, Peking Union Medical College Hospital, Chinese Academy of Medical Science, Beijing, China.

## Abstract

Reports of hypertrophic pachymeningitis associated with myeloperoxidase-antineutrophil cytoplasmic antibody (MPO-ANCA) localized exclusively in the spine were quite rare. Two cases of ANCA-associated systemic vasculitis (AASV) presenting with hypertrophic spinal pachymeningitis (HSP) causing low back pain and numbness are described. Two patients showed prominent systemic and local inflammatory reactions manifested as fever, elevated levels of erythrocyte sedimentation rate and C-reactive protein, and markedly increased levels of total protein of cerebrospinal fluid. The gadolinium (Gd)-enhanced T1-weighted magnetic resonance imaging scan of spinal cord demonstrated diffuse spinal dura matter thickening. Additionally, simple microscopic hematuria was found in 1 case suggestive of renal involvement and the other 1 complicated with interstitial lung disease. Then, a diagnosis of HSP secondary to AASV was made. Combination therapy of corticosteroids and cyclophosphamide produced a rapid improvement in the clinical symptoms and laboratory parameters. Followed up for 6 months, 1 case relapsed when the dosage of prednisone was tapered to 10 mg daily. Since the patient refused rituximab-based regimen, an immunosuppressive triple-therapy (corticosteroid, cyclophosphamide, and azathioprine) was initiated and brought control of the disease during the subsequent 6 months of follow-up.

HSP is a relatively rare form of central nervous system involvement of AASV. Early recognition and intervention are of great significance since the pathogenesis of HSP starts with an inflammatory and fibrosing process.

## INTRODUCTION

Hypertrophic pachymeningitis (HP) is a rare disorder characterized by marked inflammatory hypertrophy of the dura mater, with subsequent neurological deficits as a result of the compression of adjacent structures, which mostly involves the intracranial dura. The spinal form of HP is rather rarer. The etiologies of this disease are various which can be attributed to several disorders: infections such as syphilis,^1^ tuberculosis,^2^ human T-lymphotropic virus type 1,^3^ and fungal infection^4^; neoplastic diseases including spinal meningioma,^5^ lymphoma,^6^ heavy-chain disease^7^; trauma; autoimmune diseases including IgG4-related disease,^8^ sarcoidosis,^9^ etc.; or a combination of the disorders. Those patients with unknown etiology are classified as idiopathic hypertrophic spinal pachymeningitis (HSP). The association between ANCA and HP has been described in recent years; however, most of the cases involved the intracranial dura mater, case reports of HSP were extremely rare. In this study, we report 2 cases of ANCA-associated systemic vasculitis (AASV) presenting with HSP, and this is the first literature review of the clinical features, diagnosis, and management of this disease.

## CASE 1

A 26-year-old woman was admitted in August 2014 because of general malaise, fever, bloody nasal discharge, and persistent neck and back pain over a 4-month period. In 2005, she was diagnosed with Graves’ disease and treated with propylthiouracil (PTU) for 9 years.

**FIGURE 1:**
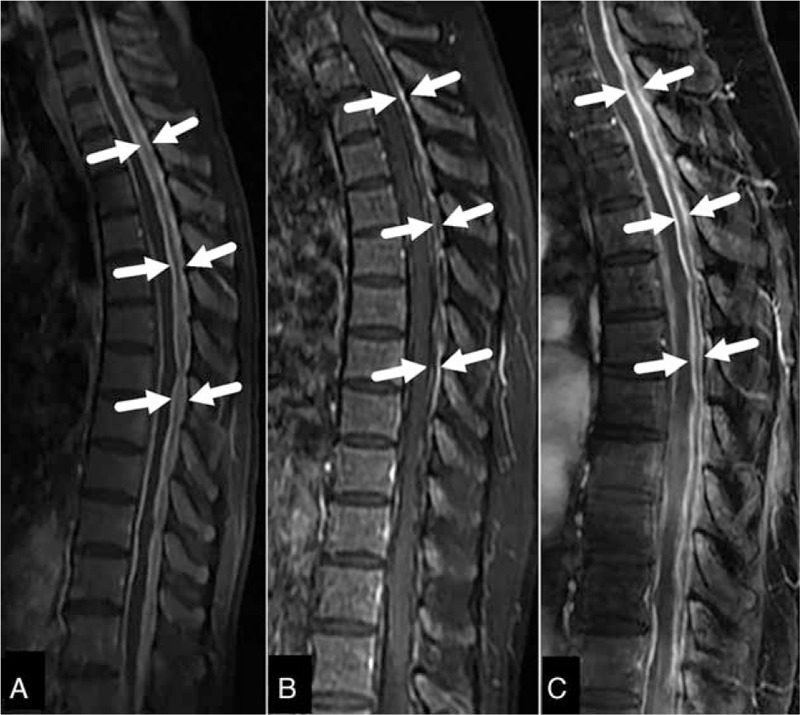
Case 1. Sagittal Gd (gadolinium)-enhanced T1-weighted magnetic resonance images (MRIs). (A) Images taken in August 2014, demonstrating diffusely thickening of dura mater at C6 and Th10 level with marked enhancement (arrow). (B) Images taken in November 2014, showing that the thickened dura mater significantly improved after immunosuppressive therapy (arrow). (C) Images taken in February 2015, demonstrating recurrence of HSP compared with previous MRI (B) (arrow).

On admission, there were no abnormal vital signs except for pyrexia (38°C). Thyroid examination showed mild enlargement and spine movement was restricted. There were no characteristic neurological findings. The laboratory test results were as follows: WBC 5.56 × 10^9^/L (normal range 4–10), hemoglobin 102 g/L (110–150), and platelets 309 × 10^9^/L (100–300). Urinalysis showed mild microscopic hematuria and urinary sediment was otherwise normal. Both renal and liver function parameters were within the normal ranges. Serum levels of free triiodothyronine (FT3), free tetraiodothyronine (FT4), and thyroid stimulating hormone (TSH) were 6.35 ng/dL (1.80–4.10), 2.400 ng/dL (0.81–1.89), and 0.017 μIU/mL (0.38–4.34), respectively. The tests of thyroglobulin antibody (TG-Ab), thyroid peroxidase antibody (TPO-Ab), and thyrotropin receptor antibodies (TR-Ab) were negative. The erythrocyte sedimentation rate (ESR) was elevated at 119 mm/hr (0–20) and hypersensitive C-reactive protein level (hsCRP) was elevated at 190 mg/L (<3). The total IgG, IgG4, IgA, and IgM levels were normal. The complement fraction C3 was 1.885 g/L (0.73–1.46) and C4 was normal. Antinuclear antibody (ANA) tests were negative. The rheumatoid factor (RF) level was 48.8 IU/mL (0–20). The serum level of myeloperoxidase (MPO)-antineutrophil cytoplasmic antibody (ANCA) was elevated at 92 RU/mL (<20) and proteinase 3 (PR3)-ANCA was negative. Examination of the cerebrospinal fluid (CSF) revealed normal CSF pressure and the total protein level of CSF was markedly elevated to 42 g/L (0.15–0.45) and a cell count of 30 per μL (lymphocytes 30). A cytological examination did not show any malignant cells, and bacterial, fungal, mycobacteria, and viral examinations were all negative in the sera as well as in the CSF. A gadolinium (Gd)-enhanced magnetic resonance image (MRI) scan of spinal cord demonstrated diffuse spinal dura matter thickening between the spinal level at the 6th cervical (C6) and 10th thoracic (Th10) cord (Fig. 1A). Electrocardiography and computed tomography (CT) scan detected no remarkable findings. Nasal involvement could not be substantiated either on nasal CT scan or otorhinolaryngological evaluation despite complaint of bloody nasal discharge. Then, spinal dura biopsy was planned; however, the patient and her families refused the biopsy procedure after a careful consideration about the risk and benefit.

In a series of 92 cases with AASV caused by antithyroid drugs in 2009,^10^ the time to onset of PTU-induced AASV varied (median time, 39 months; range, 1–132 months) after starting PTU treatment. The spectrum of manifestations was wide and ranged from less specific syndromes to single organ involvement and life-threatening vasculitis^10^, among which kidney involvement was reported to be the most frequent finding (38.2%), which was followed by respiratory tract involvement (19.0%), arthralgia and myalgia (18.4%), skin involvement (13.8%), and involvement of brain and nerves (2.0%). Considering the temporal relation of newly onset AASV to use of PTU and kidney involvement being the most frequent finding, the diagnosis of PTU-induced AASV was preferred.

PTU was discontinued immediately and no further anti-thyroid treatment was needed because of low thyroid ^131^Iodine absorption rate according to the endocrinologist's advice. Moreover, pulse intravenous methylprednisolone (1 g daily) for 3 days and followed by oral prednisone (1 mg/kg per day) with gradual tapering of the dosage and intravenous cyclophosphamide (0.4 g weekly) were given. Followed up for 3 months, the symptoms improved, ESR and hsCRP returned to normal, the protein level of CSF decreased to 1.09 g/L, MPO-ANCA decreased to 69 RU/mL. The MRI findings improved significantly (Fig. 1B).

Although the prognosis was good in the majority of patients with PTU induced AASV after cessation of the drug, it was reported that in some cases the disease might be fatal or result in permanent sequelae such as chronic renal failure relying on dialysis despite of intensive immunosuppressive therapy.^11,12^ As for this case, relapse occurred in February 2015 when she was on a dose of 10 mg of prednisone daily, she was admitted to our hospital because of a recurrence of neck and back pain. On admission, her laboratory findings were as follows: WBC 8.25 × 10^9^/L, hemoglobin 132 g/L, platelets 223 × 10^9^/L. The serum hsCRP level was elevated at 57 mg/L, and the ESR was also raised at 37 mm/hr. The serum MPO-ANCA level was elevated at 59 RU/mL. The serum levels of IgG, IgA, IgM, and complement components were within the normal ranges. ANA and RF were negative. The total protein level of CSF was elevated to 7.81 g/L. MRI revealed increase of diffusely thickened dura compared with the previous MRI (Fig. 1C). A diagnosis of recurrent AASV-associated HSP was made. Rituximab-based regimen was a good option in her situation; however, she could not afford it because the costs were not covered by medical insurance in China. So an immunosuppressive triple-therapy (corticosteroid, cyclophosphamide, and azathioprine) and intrathecal injection of dexamethasone were initiated according to our experience. Her symptoms and laboratory findings were ameliorated again. Followed up for 6 months, there was no recurrence.

## CASE 2

A 62-year-old woman who presented with general malaise, anorexia, intermittent fever, and numbness of lower limbs over a 2-year period developed low back pain 5 months before being admitted to our hospital in November 2014.

In May 2012, she complained of general malaise, anorexia, and numbness of lower limbs, accompanied by pyrexia (38.9°C), and diagnosed with probable ANCA-associated vasculitis by virtue of a positive test of MPO-ANCA and elevated ESR level of 78 mm/hr and hsCRP level of 73 mg/L. The treatment with 60 mg daily of prednisolone was started and gradually tapered, combined with 10 mg daily of leflunomide, leading to clinical remission. In June 2014, she developed low back pain in addition to numbness of lower limbs and intermittent fever.

**FIGURE 2:**
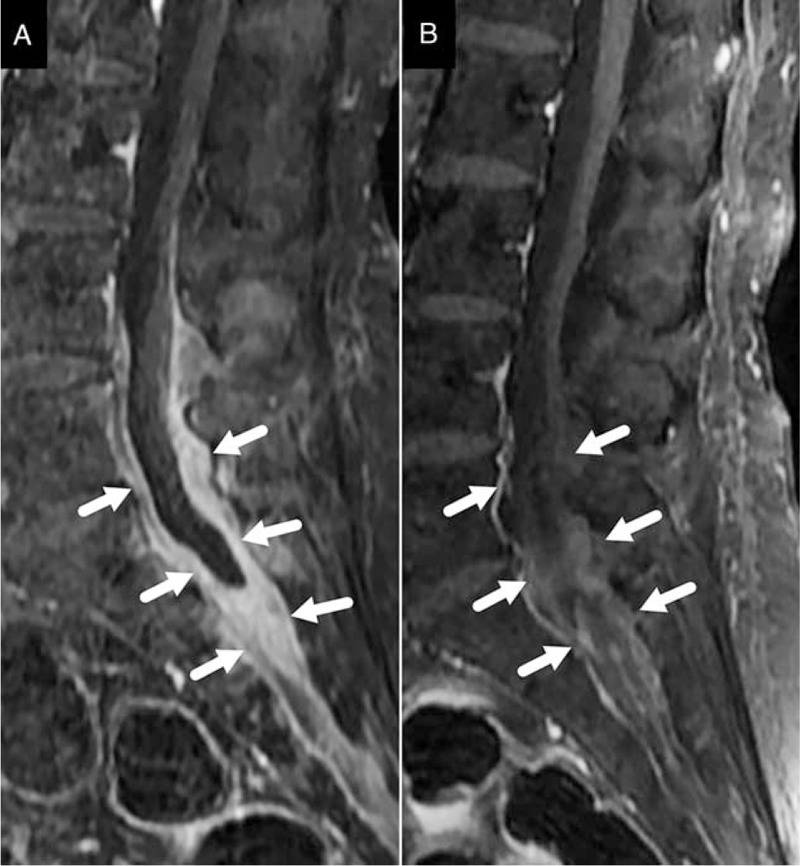
Case 2. Sagittal Gd-enhanced T1-weighted magnetic resonance images. (A) Images taken in November 2014, demonstrating diffusely thickening of dura mater at L4 and S3 level with marked enhancement (arrow). (B) Images taken in February 2015, showing that the thickened dura mater significantly improved after immunosuppressive therapy (arrow).

On admission, there were no characteristic physical or neurological findings. Laboratory data were as follows: WBC 5.52 × 10^9^/L, hemoglobin 94 g/L, and platelets 276 × 10^9^/L. Urinalysis was normal. Electrolytes, renal function, liver enzymes, thyroid tests, and angiotensin-converting enzyme were all within the normal ranges. ESR was 101 mm/hr and hsCRP was 48.94 mg/L. The serum level of interleukin-6 (IL-6) was markedly elevated to 46.6 pg/mL (<4.0). RF and MPO-ANCA were 80 IU/mL and 33 RU/mL, respectively. Other serological tests including ANA, anti-double stranded DNA antibody, anticardiolipin antibody, and PR3-ANCA were all negative. The serum levels of IgG, IgG4, IgA, IgM, and complement components were all within normal ranges. Examination of CSF revealed normal CSF pressure, with elevated protein level of 1.64 g/L and a cell count of 63 per μL (lymphocytes 54). Cytological examination of CSF showed no malignant cells, and repeated examinations of bacterial, fungal, mycobacteria, and viral were all negative in the sera as well as in CSF. Electrocardiography was normal. Interstitial lung disease in inferior lobes of both lungs was detected by high-resolution CT scan. Gd-enhanced T1-weighted MRI scan of spinal cord demonstrated diffuse spinal dura matter thickening between the spinal level at the 4th lumbar (L4) and 3rd sacral (S3) cord (Fig. 2A). Then, a diagnosis of AASV with HSP was made, and pulse intravenous methylprednisolone (1 g daily) for 3 days and followed by oral prednisone (60 mg/day) with gradual tapering of the dose and oral cyclophosphamide (0.2 g every other day) were given. Followed up for 6 months, the symptoms improved rapidly, ESR, hsCRP, and the protein level of CSF returned to normal. MPO-ANCA was undetected. The MRI findings were markedly improved (Fig. 2B).

## METHODS

Ethical approval was waived since the study was based on a review of medical records that had been obtained for clinical purposes. And the written informed consent was signed by the 2 patients. To explore the clinical and laboratory features, diagnosis, and management about ANCA-associated HSP, we searched the PubMed and Medline databases for reports of cases by using the keywords “hypertrophica,” “pachymeningitis,” “spinal cord,” “hypertrophy spinal pachymeningitis,” “antineutrophil cytoplasmic antibody,” “ANCA,” “Wegener's granulomatosis,” “microscopic polyangiitis,” “Churg Strauss,” and “vasculitis” in different combinations.

## RESULTS

We identified 10 cases described in English with detailed data.^13–22^ The general characteristics of the patients and the 2 in the present study are reviewed in Table 1. Their median age at onset was 56 years (ranged from 26 to 77 years), and it showed a female preponderance (the ratio of female to male is 10:2) which is consistent with Yokoseki's study about ANCA-associated HP^23^. The titers of ANCA were elevated in all cases. Two out of 10 cases^16,18^ were diagnosed as ANCA-associated HSP since a lack of multiple-organ involvements, and other 8 cases were diagnosed with AASV, 7 of which were classified as GPA (also known as Wegener's granulomatosis) and presented with at least 1 other organ involvements, that is, articular, renal, pulmonary, and otolaryngological. Spinal dural mater involvement was present in 9 out of 12 cases when the diagnosis of vasculitis was made. The main symptoms resulting from the compression of adjacent spinal cord were back pain, sensory/motor disturbances, and even paraplegia. Patients presented with prominent systemic and local inflammation including presence of fever (7 out of 12 cases), elevated CRP (8 out of 8 cases), and markedly elevated levels of total protein in CSF (6 out of 7 cases). Contrast-enhanced MRI of spinal dura mater was reported to be the most valuable diagnostic imaging technique and the typical imaging findings were iso- or low-intense signal on T1-weighted images and lower-intense signal on T2-weighted images which mostly affect thoracic vertebral levels in a consecutive or discrete^15^ manner and can be markedly enhanced after injection of the contrast media. Biopsy of dura mater was the gold standard diagnostic test, which may reveal fibrosis with infiltration of a variety of chronic inflammatory cells, or coexist with formation of epithelioid granulomas or vasculitis. A study from Japan^23^ showed that CXCL10, CXCL8, IL-6 were elevated in the CSF of patients with ANCA-positive HP which was suggestive of accumulation of activated macrophages and neutrophils with Th1 lymphocytes, and ectopic lymphoid neogenesis could be seen in the thickened dura mater from several patients which may play a role in initiating and maintaining immune responses.

**TABLE 1:**
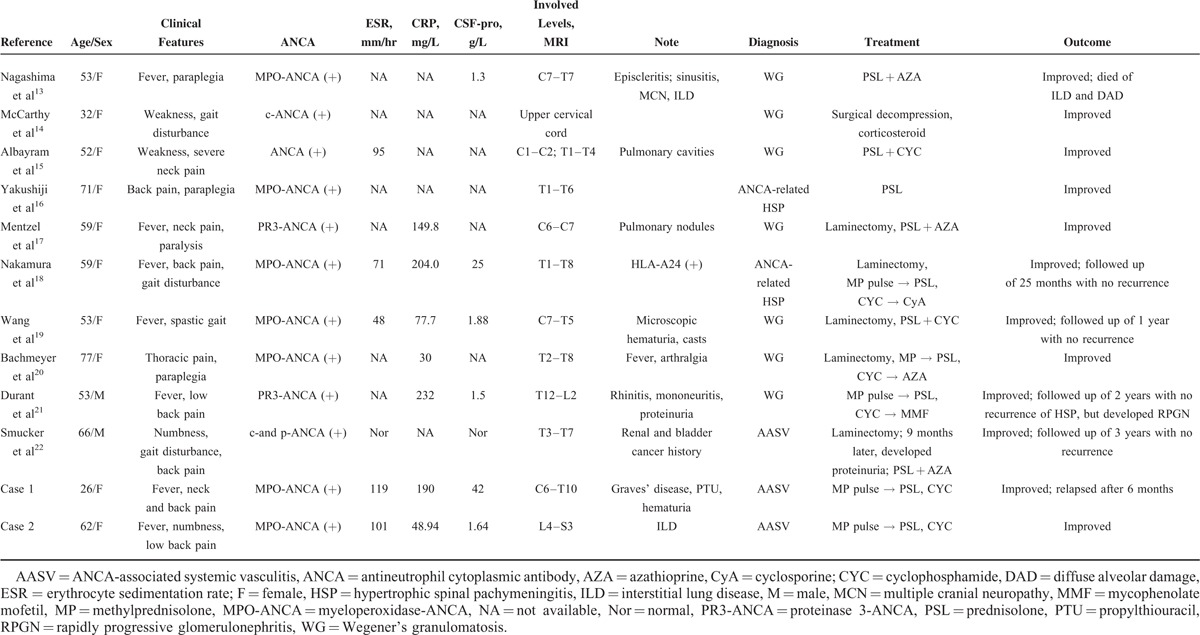
Review of Previous Reported and Present Cases With ANCA Positive HSP

Overall, all the patients improved rapidly following combination therapy of steroids and immunosuppressants; however, 1 case died of lung complications associated with GPA,^13^ another 1 developed rapidly progressive glomerulonephritis 2 years later,^21^ and case 1 relapsed after 6 months when the dosage of prednisone reduced to 10 mg/day. Of note, laminectomy may temporarily relieve the clinical symptoms, but disease could not be controlled without subsequent immunosuppressive therapy.^22^

## DISCUSSION

AASV predominantly affect small-sized blood vessels which typically presents with multiple-organ involvements including skin, kidney, respiratory tract, peripheral nerves, and gastrointestinal tract,^24^ among which HP, a form of central nervous system (CNS) involvement, is quite rare. A nationwide survey of HP from Japan^25^ showed that idiopathic HP was most common (accounts for 44%), while ANCA-associated HP, especially granulomatosis with polyangiitis (GPA), was the most frequent form of secondary HP (accounts for 34%), followed by IgG4-related HP (accounts for 8.8%). Yokoseki et al^23^ proposed that ANCA-associated HP might be a limited neurologic form of AASV.

HSP can be secondary to infections, neoplastic diseases, and autoimmune diseases and detailed differential diagnosis is essential. Firstly, HSP can be present in a variety of CNS infections with the following pathogenic microorganisms, such as *Staphylococcus*, *Aspergillus*, *Mycobacterium tuberculosis*, *Treponema pallidum*, etc.^1,2,4^ As for the 2 patients reported here, repeated examination of the above pathogenic microorganisms in both peripheral blood and CSF was all negative and subsequent good response to the therapy of glucocorticoids and immunosuppressants did not support the diagnosis of CNS infections. Secondly, neoplastic diseases such as meningioma, lymphoma, and heavy-chain disease could also result in thickening of spinal dura.^5–7^ However, CT scans of the chest, abdomen, and pelvis showed no evidence of neoplasm and cytological examination of CSF likewise showed no malignant cells. Additionally, abnormal heavy or light chain and monoclonal brand were not detected with serum protein electrophoresis and immunofixation electrophoresis. As a consequence, malignancies were excluded. Thirdly, autoimmune diseases like IgG4-related disease and systemic lupus erythematosus were not considered since serum detection of ANA was negative and serum level of IgG4 was normal.

Therefore, the diagnosis of AASV was established based on the presence of general malaise, anorexia, fever, multiple-organ involvements mainly manifested as HSP and ILD or microscopic hematuria, markedly elevated inflammatory parameters including ESR, CRP and IL-6, and positive MPO-ANCA serology. Though vasculitis was not proven histologically, other etiologies mentioned above had been ruled out, and subsequently good response to the immunosuppressive therapy confirmed the diagnosis. In patient 1, PTU induced AASV was strongly suspected based on her past medication history and the clinical resolution after cessation of the drug and treatment with immunosuppressants.

Combination therapy of glucocorticoid and cyclophosphamide has been the standard regimen for remission induction for nearly 4 decades and this regimen has brought patients with severe AASV a better disease control and temporary remission. Because of multiple adverse effects of cyclophosphamide and lengthy courses of glucocorticoid treatment, recent results of Rituximab in ANCA-Associated Vasculitis (RAVE) trial demonstrated that rituximab was as effective as cyclophosphamide for the induction of remission in patients with severe AASV.^26^ Moreover, the rituximab-based regimen was superior in patients with relapsing disease at 6 months.^27,28^ However, the efficacy was unclarified owing to the rarity of CNS involvement especially HSP in AASV. Holle et al^29^ compared the efficacy of rituximab for the treatment of granulomatous and vasculitic manifestations of 59 patients with refractory GPA. Interestingly, complete or partial remission was achieved for 90% of vasculitic but only 58% of granulomatous manifestations, and for patients with pachymeningitis, 50% responded, with only 8.3% achieving complete remission. Thus, their results suggest that the response rate to rituximab treatment depends on the proportion of vasculitis and granulomatous inflammation, respectively, in the spinal dura lesions.

When the diagnosis of HSP is made by a spinal surgeon at the first visit, attention should be paid to whether there are systemic signs such as fever, multiple-organ involvement, and elevated levels of inflammatory markers like ESR and CRP since these are predominant features of HSP patients with AASV or other systemic diseases. So baseline screening of ANCA, immunoglobulin G, and its subclass or the spectrum of ANA are recommended and multidisciplinary collaboration is needed. Of note, spinal dura biopsy is the gold standard of diagnosis and meaningful for differential diagnosis.

## CONCLUSIONS

To conclude, HSP is a relatively rare form of CNS involvement of AASV, especially in GPA patients. When patients with AASV complained of backache or paralysis of the legs, HSP should be suspected. Conversely, patients diagnosed with HSP should be screened with ANCA test or other autoimmune diseases. Although it might be underdiagnosed on account of the lack of histologically evidence, contrast-enhanced MRI is essential for clinical diagnosis and good response to subsequent combinational therapy of steroids and immunosuppressants could be helpful for the confirmation of the diagnosis. Early recognition and intervention are of great significance since the pathogenesis of HSP starts with an inflammatory and fibrosing process; some patients may require surgical decompression to prevent irreversible neurological impairment if there is no response or relapse after the traditional therapy.

## References

[1] Vale TC Moraes TE Lara A Hypertrophic cervical spinal cord pachymeningitis due to *Treponema pallidum* infection. *Neurol Sci* 2012; 33:359–362. 21863268 10.1007/s10072-011-0738-6

[2] Tariq R Ahmed R Tuberculous hypertrophic pachymeningitis presenting as visual blurring and headaches. *J Pak Med Assoc* 2012; 62:966–968. 23139987

[3] Kawano Y Kira J Chronic hypertrophic cranial pachymeningitis associated with HTLV-I infection. *J Neurol Neurosurg Psychiatry* 1995; 59:435–437. 7561926 10.1136/jnnp.59.4.435 PMC486083

[4] Murai H Kira J Kobayashi T Hypertrophic cranial pachymeningitis due to *Aspergillus flavus*. *Clin Neurol Neurosurg* 1992; 94:247–250. 1327616 10.1016/0303-8467(92)90097-m

[5] Nakase H Ohnishi H Watabe Y Lateral approach for anterior thoracic spinal lesions. *Neurol Med Chir (Tokyo)* 1994; 34:530–533. 7526238 10.2176/nmc.34.530

[6] Hsu HT Hsu SS Chien CC Teaching NeuroImages: idiopathic hypertrophic spinal pachymeningitis mimicking epidural lymphoma. *Neurology* 2015; 84:e67–e68. 25732370 10.1212/WNL.0000000000001314

[7] Yunokawa K Hagiyama Y Mochizuki Y Hypertrophic spinal pachymeningitis associated with heavy-chain disease. Case report. *J Neurosurg Spine* 2007; 7:459–462. 17933324 10.3171/SPI-07/10/459

[8] Ezzeldin M Shawagfeh A Schnadig V Hypertrophic spinal pachymeningitis: idiopathic vs. IgG4-related. *J Neurol Sci* 2014; 347:398–400. 25454650 10.1016/j.jns.2014.10.012

[9] Marangoni S Argentiero V Tavolato B Neurosarcoidosis. Clinical description of 7 cases with a proposal for a new diagnostic strategy. *J Neurol* 2006; 253:488–495. 16283095 10.1007/s00415-005-0043-5

[10] Noh JY Yasuda S Sato S Clinical characteristics of myeloperoxidase antineutrophil cytoplasmic antibody-associated vasculitis caused by antithyroid drugs. *J Clin Endocrinol Metab* 2009; 94:2806–2811. 19491223 10.1210/jc.2008-2700

[11] Batchelor N Holley A A fatal case of propylthiouracil-induced ANCA-positive vasculitis. *MedGenMed* 2006; 8:10. 17415292 PMC1868332

[12] Chen YX Yu HJ Ni LY Propylthiouracil-associated antineutrophil cytoplasmic autoantibody-positive vasculitis: retrospective study of 19 cases. *J Rheumatol* 2007; 34:2451–2456. 17985400

[13] Nagashima T Maguchi S Terayama Y P-ANCA-positive Wegener's granulomatosis presenting with hypertrophic pachymeningitis and multiple cranial neuropathies: case report and review of literature. *Neuropathology* 2000; 20:23–30. 10935433 10.1046/j.1440-1789.2000.00282.x

[14] McCarthy PJ Arend WP Kleinschmidt-DeMasters BK May 2001: 32 year old female with dural mass encircling cervical spinal cord. *Brain Pathol* 2001; 11:483–484.487. 11556695 10.1111/j.1750-3639.2001.tb01090.x PMC8098198

[15] Albayram S Kizilkilie O Adaletli I MR imaging findings of spinal dural involvement with Wegener granulomatosis. *Am J Neuroradiol* 2002; 23:1603–1606. 12372756 PMC7976796

[16] Yakushiji Y Kurohara K Toda S A case of hypertrophic spinal pachymeningitis associated with MPO-ANCA. *Rinsho Shinkeigaku* 2002; 42:873–877. 12710088

[17] Mentzel HJ Neumann T Fitzek C MR imaging in Wegener granulomatosis of the spinal cord. *Am J Neuroradiol* 2003; 24:18–21. 12533321 PMC8148975

[18] Nakamura T Hirakawa K Higashi S CD8^+^ T lymphocytes infiltrate predominantly in the inflammatory foci of MPO-ANCA-positive thoracic hypertrophic pachymeningitis in a patient with HLA-A24. *Mod Rheumatol* 2007; 17:75–80. 17278028 10.1007/s10165-006-0537-8

[19] Wang DC Wei JW Liu JH The upper thoracic spinal cord compression as the initial manifestation of Wegener's granulomatosis: a case report. *Eur Spine J* 2007; 16 Suppl 3:296–300. 17294055 10.1007/s00586-007-0318-x PMC2148087

[20] Bachmeyer C Cervera P Marro B Thoracic spinal cord compression indicating Wegener's granulomatosis in a patient with a previous presumptive diagnosis of microscopic polyangiitis. *Joint Bone Spine* 2007; 74:382–384. 17587626 10.1016/j.jbspin.2006.10.008

[21] Durant C Martin J Godmer P Exceptional osseous and meningeal spinal localization of ANCA-associated granulomatous vasculitis with hypertrophic spinal pachymeningitis. *J Neurol* 2011; 258:1172–1173. 21210138 10.1007/s00415-010-5886-8

[22] Smucker JD Ramme AJ Leblond RF Hypertrophic spinal pachymeningitis with thoracic myelopathy: the initial presentation of ANCA-related systemic vasculitis. *J Spinal Disord Tech* 2011; 24:525–532. 21436740 10.1097/BSD.0b013e3182067abf

[23] Yokoseki A Saji E Arakawa M Hypertrophic pachymeningitis: significance of myeloperoxidase anti-neutrophil cytoplasmic antibody. *Brain* 2014; 137:520–536. 24271323 10.1093/brain/awt314

[24] Jennette JC Falk RJ Bacon PA 2012 revised International Chapel Hill Consensus Conference Nomenclature of Vasculitides. *Arthritis Rheum* 2013; 65:1–11. 23045170 10.1002/art.37715

[25] Yonekawa T Murai H Utsuki S A nationwide survey of hypertrophic pachymeningitis in Japan. *J Neurol Neurosurg Psychiatry* 2014; 85:732–739. 24273222 10.1136/jnnp-2013-306410

[26] Stone JH Merkel PA Spiera R Rituximab versus cyclophosphamide for ANCA-associated vasculitis. *N Engl J Med* 2010; 363:221–232. 20647199 10.1056/NEJMoa0909905 PMC3137658

[27] Specks U Merkel PA Seo P Efficacy of remission-induction regimens for ANCA-associated vasculitis. *N Engl J Med* 2013; 369:417–427. 23902481 10.1056/NEJMoa1213277 PMC5953195

[28] Miloslavsky EM Specks U Merkel PA Rituximab for the treatment of relapses in antineutrophil cytoplasmic antibody-associated vasculitis. *Arthritis Rheumatol* 2014; 66:3151–3159. 25047592 10.1002/art.38788 PMC4229846

[29] Holle JU Dubrau C Herlyn K Rituximab for refractory granulomatosis with polyangiitis (Wegener's granulomatosis): comparison of efficacy in granulomatous versus vasculitic manifestations. *Ann Rheum Dis* 2012; 71:327–333. 22021864 10.1136/ard.2011.153601

